# Modeling bispecific monoclonal antibody interaction with two cell membrane targets indicates the importance of surface diffusion

**DOI:** 10.1080/19420862.2016.1178437

**Published:** 2016-04-20

**Authors:** Bram G. Sengers, Sean McGinty, Fatma Z. Nouri, Maryam Argungu, Emma Hawkins, Aymen Hadji, Andrew Weber, Adam Taylor, Armin Sepp

**Affiliations:** a Bioengineering Science Research Group, Faculty of Engineering and the Environment, and Institute for Life Sciences, University of Southampton, Southampton, UK; b Division of Biomedical Engineering, Glasgow University, Glasgow, UK; c Laboratoire de Modélisation Mathématiques et Simulation Numérique, Faculté des Sciences, Université Badji-Mokhtar, Annaba, Algeria; d Department of Bioengineering, Imperial College London, London, UK; e Department of Mathematics, University of Surrey, Guildford, UK; f Pharmaceutic Mineral Chemistry Laboratory, Université Badji-Mokhtar, Annaba, Algeria; g Drug Metabolism and Pharmacokinetics, GlaxoSmithKline Plc., King of Prussia, PA, USA; h Respiratory DPU, GlaxoSmithKline Plc., Stevenage, UK; i Drug Metabolism and Pharmacokinetics, GlaxoSmithKline Plc., Stevenage, UK

**Keywords:** Affinity, avidity, bispecific antibody, diffusion, Monte Carlo, modeling, specificity, simulation, therapeutic

## Abstract

We have developed a mathematical framework for describing a bispecific monoclonal antibody interaction with two independent membrane-bound targets that are expressed on the same cell surface. The bispecific antibody in solution binds either of the two targets first, and then cross-links with the second one while on the cell surface, subject to rate-limiting lateral diffusion step within the lifetime of the monovalently engaged antibody-antigen complex. At experimental densities, only a small fraction of the free targets is expected to lie within the reach of the antibody binding sites at any time. Using ordinary differential equation and Monte Carlo simulation-based models, we validated this approach against an independently published anti-CD4/CD70 DuetMab experimental data set. As a result of dimensional reduction, the cell surface reaction is expected to be so rapid that, in agreement with the experimental data, no monovalently bound bispecific antibody binary complexes accumulate until cross-linking is complete. The dissociation of the bispecific antibody from the ternary cross-linked complex is expected to be significantly slower than that from either of the monovalently bound variants. We estimate that the effective affinity of the bivalently bound bispecific antibody is enhanced for about 4 orders of magnitude over that of the monovalently bound species. This avidity enhancement allows for the highly specific binding of anti-CD4/CD70 DuetMab to the cells that are positive for both target antigens over those that express only one or the other We suggest that the lateral diffusion of target antigens in the cell membrane also plays a key role in the avidity effect of natural antibodies and other bivalent ligands in their interactions with their respective cell surface receptors.

## Abbreviations

M&S: modeling and simulation
ODE: ordinary differential equation
K_d_: dissociation equilibrium constant
k_a_: association rate constant
k_d_: dissociation rate constant
mAb: monoclonal antibody
MFI: mean fluorescence intensity
EGFR: epidermal growth factor receptor
IGF1R: insulin-like growth factor 1 receptor

## Introduction

Mathematical modeling and simulation (M&S) is frequently used for testing hypotheses and gaining quantitative insight into the dynamics of complex systems. This is especially relevant for the understanding and prediction of properties of therapeutic bispecific monoclonal antibodies (mAbs), of which many different formats are in development.^1^ These engineered proteins do not exist naturally, but are expected to allow improved drug targeting and efficacy compared with the wild-type mAbs. As a result, there is an increasing number of bispecific mAbs entering clinical trials, with targets ranging from cytokines to membrane-expressed receptors.^2-4^

While bispecific mAb interactions with 2 different soluble targets can be expected to be independent from each other, the situation is different when both targets are expressed on the surface of the same cell. In this case, efficient binding can be observed at antibody concentrations well below the values of individual dissociation constants (K_d_) for either interaction, as was convincingly demonstrated recently by Mazor et al.^5,6^ who studied the reactivity of a series of anti-CD4/CD70 DuetMab bispecific mAbs against cells expressing both targets or just one or the other. Even more, these experimental results suggested that, at the concentrations tested, the DuetMab fraction bound to dual-positive cells was always engaging both antigens at the same time, despite CD4 and CD70 surface density being too low for both antigens to be simultaneously within the reach of the antigen-binding sites on the two arms of the mAb.

No quantitative analysis was given in Mazor et al.^5^ to describe and predict how the bispecific mAb used interacted with the two cell-surface antigens, which are not known to interact with each other and are expressed at densities for which the average distance of ∼45 nm between the individual molecules far exceeds the approximately 9 nm reach of the antibody antigen-binding arms. Given the growing interest in IgG-like bispecific antibodies in the biopharmaceutical industry, as evidenced by advances in heterodimerization technology and the various alternative formats being considered (e.g., Genentech “knob-into-holes”,^7^ κλ-bodies from Novimmune,^8^ and others^9^), we developed a quantitative M&S framework to address this issue and compared the predictions with the experimental results published by Mazor et al.^6^ in the expectation that the principles described can also be extended to more complex cases in future.

**Figure 1.**
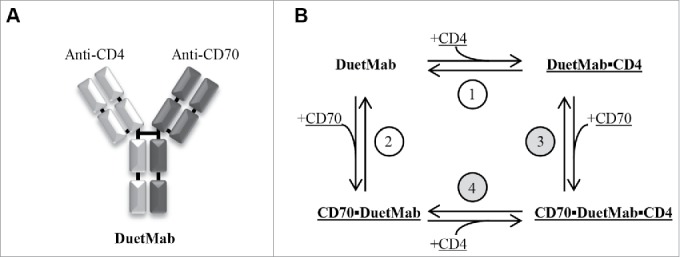
The anti-CD4/CD70 DuetMab described by Mazor et al.^5,6^ and our proposed reaction scheme. (A) A DuetMab is monovalent for either of the antigens and shares the overall structure with natural IgG. Appropriate pairing of the 2 different pairs of heavy and light chains is achieved through proprietary guiding mutations. (B) DuetMab in solution binds either of the 2 cell surface antigens to form a surface-bound binary complex (reactions 1 and 2), which then cross-links with the second target to form a ternary complex (reactions 3 and 4). Surface-bound species are underlined. Reactions involving surface-bound species only are shaded.

We analyze an IgG-like bispecific mAb that binds first either of the two antigens on the cell surface to form a reversible monovalently bound binary complex. The remaining free binding site of the mAb in the binary complex then binds a free second target molecule on the cell surface to form a ternary complex (Fig. 1).

In the first step, one of the reactants is in solution and the other one is on the surface, while in the second step both reactants are surface-bound. We used two alternative approaches to model the reaction scheme shown in Fig. 1B. The ordinary differential equation (ODE) model is based on the well-stirred assumption. Monte Carlo (MC) simulations are slower, but are not constrained by well-stirred approximation and explicitly define the role for diffusion and Brownian motion. Both models can be formulated using experimentally measurable parameters only and do not rely on empirical curve-fitting.

The resulting models can be useful for pharmacokinetic and pharmacodynamic modeling of novel drug candidates and targets, as well as understanding bivalent ligand interaction with two cell membrane receptors in general. We also consider the implications for monospecific and bispecific mAbs binding to homo- and heterodimeric membrane-bound targets, and consider the advantages and disadvantages of applying a combination of monospecific mAbs vs respective bispecific variants in the case of cell surface-expressed targets.

## Results

**Figure 2.**
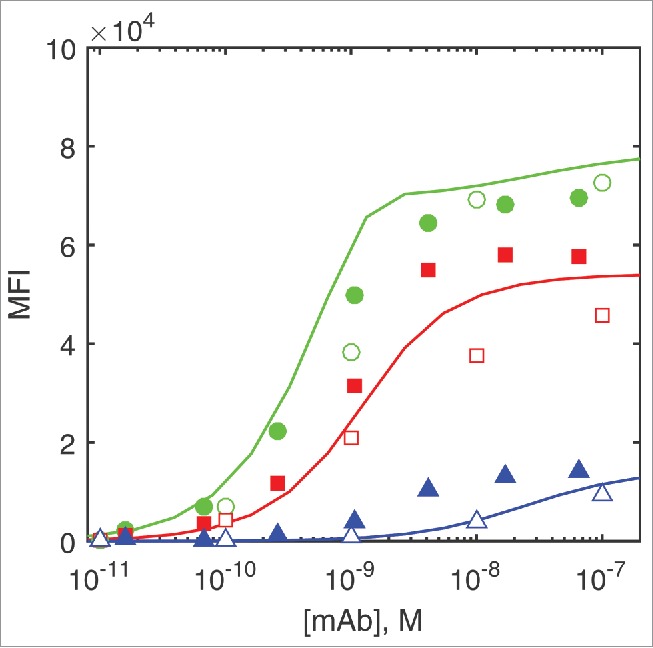
The experimentally measured and predicted binding of the parent DuetMab in flow cytometry assay. Filled circles, squares and triangles are experimental data measured by Mazor et al.^6^ for CD4^+^/CD70^+^, CD4^+^/CD70^−^ and CD4^−^/CD70^+^ cells, respectively. Empty symbols represent Monte Carlo simulation results and the lines are the ODE simulation results for the same cell types, respectively.

Mazor et al.^6^ analyzed the binding activity of the parent DuetMab that had high affinity for CD4 (K_d_ = 0.9 nM) and low affinity for CD70 (K_d_ = 25 nM) using cells expressing both antigens at the same time or just one or the other. In agreement with the experimental data, both ODE and Monte Carlo models predict higher binding of the DuetMab to dual-positive CD4^+^/CD70^+^ and single-positive CD4^+^/CD70^−^ cells than to the CD4^−^/CD70^+^ ones (Fig. 2).

**Figure 3.**
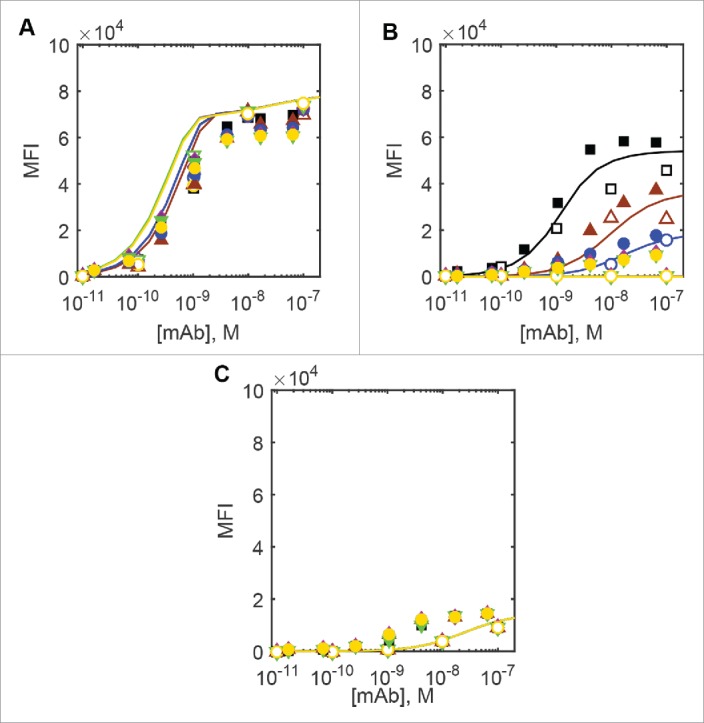
Experimental and predicted flow cytometric cell binding curves for the parent and modified DuetMabs. Black squares, brown triangles pointing up, blue circles, green triangles pointing down, purple diamonds and yellow circles denote parent DuetMab and the anti-CD4 affinity attenuated variants VkY92A, VkY91A, VkR95A, VkR95A+VhD97A and VkR94A+VhD97A. Filled symbols denote the experimental data measured by Mazor et al.,^6^ and empty symbols Monte Carlo simulation results. Lines denote ODE simulation results in the same order. A) dual-positive CD4^+^/CD70^+^ cells, B) single-positive CD4^+^/CD70^−^ cells and C) single-positive CD4^−^/CD70^+^ cells.

In an effort to improve DuetMab preferential binding to dual-positive cells, Mazor et al.^6^ constructed a series of DuetMab variants with progressively attenuated affinities for CD4, while keeping the binding for CD70 unchanged. Surprisingly, strong binding of DuetMab variants even at 1 nM concentrations to CD4^+^/CD70^+^ dual-positive cells was virtually unaffected, while all but non-specific binding was eliminated on single-positive CD4^+^/CD70^−^ and CD4^−^/CD70^+^ cells. We find that both ODE and Monte Carlo models adequately capture these experimental findings, when rapid first-order dissociation of monovalently bound DuetMab species according to their dissociation rate constant from the single-positive CD4^+^/CD70^−^ and CD4^−^/CD70^+^ cells is taken into account, as shown on Figs. 3(A, B and C).

We therefore conclude that both ODE and MC approaches are in agreement with the experimental data described by Mazor et al.,^6^ as well as with each other and hence both approaches can be used to gain further insight into the DuetMab-type bispecific antibody interactions with cell surface targets.

**Figure 4.**
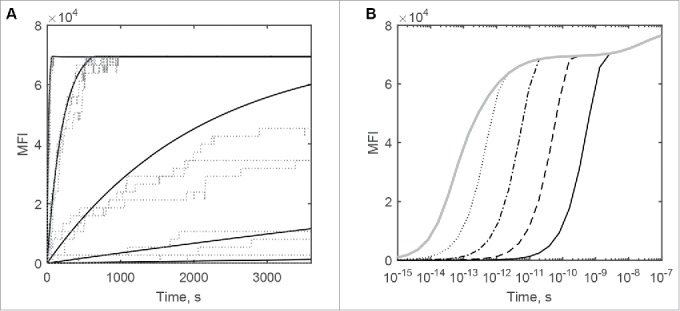
The predicted effect of time on the flow cytometric binding of the parent DuetMab to CD4^+^/CD70^+^ dual-positive cells. (A) One-hour incubation with the antibody. Solid lines from left to right are ODE simulations at 100 nM, 10 nM, 1 nM, 0.1 nM and 0.01 nM constant DuetMab concentrations. Dotted lines denote respective Monte Carlo simulations. (B) Extended incubation time is predicted to increase DuetMab binding at lower concentrations. Black solid, dashed, dot-dashed, dotted and gray solid lines denote the flow cytometric binding curve simulations for 1 h, 10 h, 100 h, 1000 h and 10000 h incubation times.

Both ODE and MC models suggest that steady-state equilibrium is only achieved during the one hour experimental incubation step at DuetMab concentrations above 1 nM. Progressively longer incubation times at lower concentrations would increase the amount of bound antibody to an extent, as shown in Fig. 4. While very long incubation times are not experimentally feasible for the labeling of live cells at low temperatures, this would be relevant in a therapeutic setting, given the long in vivo half-life of antibodies. It should, however, be noted that in this case target internalization kinetics (suppressed in the cell labeling experiment due to the low temperature and omitted in the model) would also need to be taken into account.

**Figure 5.**
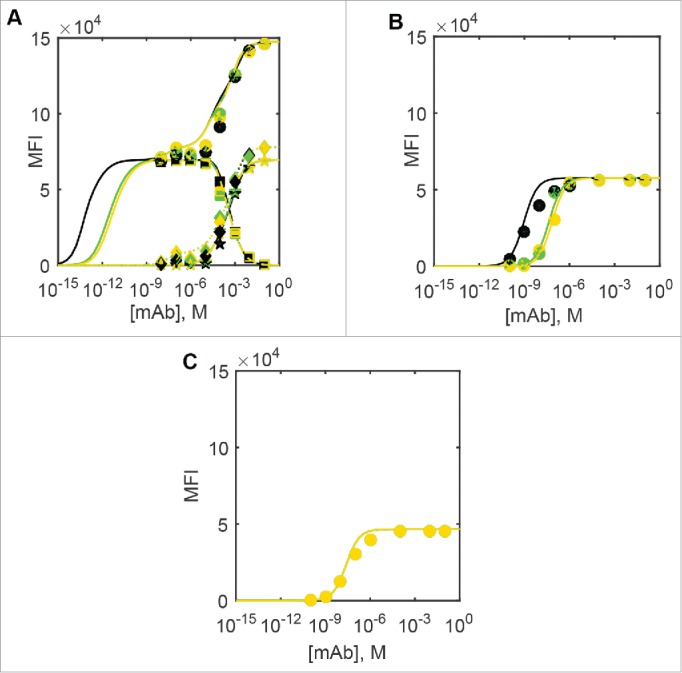
Predicted steady-state cell surface concentration of DuetMab in MFI units. Black: parent DuetMab, green: Vk(R95A) variant and yellow: Vk(R94A)+VhY99A variant. ODE predictions (10000 h simulated incubation time), solid lines: total antibody, dashed lines: cross-linked antibodies, dash-dot: CD4-bound antibody, dotted lines: CD70-bound antibody. Monte Carlo simulation results shown at DuetMab>10 nM (where steady state was achieved at 1h or less). Circles: total antibody, squares: cross-linked antibodies, diamonds: CD4-bound antibody, pentagrams: CD70-bound antibody. (A) Dual-positive CD4^+^/CD70^+^ cells, (B) single-positive CD4^+^/CD70^−^ cells, (C) single-positive CD4^−^/CD70^+^ cells.

We next used ODE and Monte Carlo models (where feasible) to consider the relative surface concentrations of binary and ternary complexes of the DuetMab-like bispecific mAb as a function of an extended antibody concentration range in steady-state equilibrium conditions. The modeling results for three different DuetMabs are shown in Fig. 5 in mean fluorescence intensity (MFI) cell surface concentration units for easier comparison purposes: the parent molecule, the Vk(R95A) variant where affinities for both CD4 and CD70 are nearly equal and the weakest CD4-binding Vk(R94A)+VhY99A variant. In this case, the dissociation step applied in Fig. 2–3 has been omitted to show the predicted steady-state equilibrium surface concentration values that would be relevant in a therapeutic setting, again in MFI units for ease of comparison. In the case of dual-positive CD4^+^/CD70^+^ cells, as the DuetMab concentration increases to about 50 nM, target cross-linking is expected to take place first, with monovalently CD70-bound antibody species starting to accumulate only after all available CD4 has been sequestered into ternary complexes. This is in agreement with the experimental observations by Mazor et al.,^5,6^ who did not find any monovalently bound DuetMab on the dual-positive CD4^+^/CD70^+^ cells. At antibody concentrations of ∼1 µM (approximate maximum plasma concentration after therapeutically relevant 10 mg/kg dose) and higher, the reaction between a free surface target and a volume phase bispecific antibody increasingly outcompetes target cross-linking on the cell surface. This is expected to result in the accumulation of binary complexes at the expense of the ternary ones, but, due to the antibody concentrations required, is unlikely to be experimentally relevant. In the case of single-positive cells, the binding of the DuetMab is expected to follow simple monovalent mass action kinetics according to their respective K_d_ values for either antigen, as shown in Figs. 5B-C.

Of special interest here are the DuetMab equilibrium binding curves to the single- and dual-positive cells, where the latter allows both antigen-binding arms of the antibody to interact with cell-surface antigens. Bivalent attachment of the bispecific antibody to the targets adds about 4 orders of magnitude to the effective binding potency of the molecule relative to the monovalently bound one.

**Figure 6.**
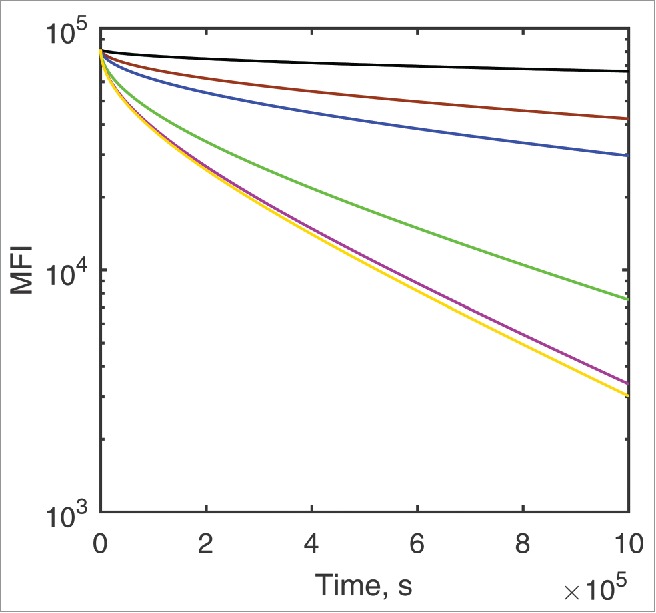
ODE-predicted time course in surface concentration MFI units for the dissociation of parent DuetMab (black) and the anti-CD4 affinity attenuated variants VkY92A (brown), VkY91A (blue), VkR95A (green), VkR95A+VhD97A (purple) and VkR94A+VhY99A (yellow) from stoichiometrically cross-linked targets on the dual-positive CD4^+^/CD70^+^ cells.

Finally, using the ODE model we also analyzed the dissociation kinetics of bivalently bound DuetMab variants from dual-positive CD4^+^/CD70^+^ cells, starting from the saturating cross-linked ternary surface complex with no antibody in solution. Unsurprisingly, while the dissociation reaction is faster for the more weakly binding variants, it happens very slowly compared with the expected monovalent dissociation curves, and it does not follow first order kinetics, getting progressively slower as the bound fraction decreases (Fig. 6).

The expected dissociation half-lives are very long and exceed t_½_≈40h even for the weakest-binding VkR94A+VhD97A variant, which from the respective single-positive cells would be expected to dissociate within minutes according to first-order kinetics (Table 1).

**Table 1.** Kinetic constants used for modeling that are taken from Mazor et al.^6^ or derived.

	CD4				CD70			
DuetMab	k_1_×10^5^ 1/(M s)	k_−1_×10^−3^ 1/s	t_−1½_ s	^1^k_−4_×10^−3^ 1/s	k_2_ ×10^5^ 1/M s)	k_−2_×10^−3^ 1/s	t_½_s	k_3_×10^13^ dm^2^/(mol s)
parent	2.8	0.26	2665	0.19	2.0	4.9	141	2.4
VkY92A	2.0	1.9	365	1.9	2.0	4.9	141	2.4
VkY91A	2.8	4.7	147	3.3	2.0	4.9	141	2.4
VkR95A	5.4	23	30	8.5	2.0	4.9	141	2.4
VkR95A+VhD97A	5.6	35	20	12.5	2.0	4.9	141	2.4
VkR94A+VhY99A	5.2	36	19	13.8	2.0	4.9	141	2.4

1 Calculated according to Equation 10.

## Discussion

Using the simple reaction scheme outlined on Fig. 1B, and only relying on experimentally measurable parameters with well-defined biochemical/biophysical meaning, we have described the data reported by Mazor et al.,^6^ who, in demanding and complex experiments, studied the binding of a series of anti-CD4/CD70 DuetMabs to three different types of cells in vitro. The key aspect of our approach, irrespective of whether ODE or Monte Carlo methods were used, is the appreciation that the cross-linking reaction is likely to take place between two surface-bound molecules with reduced degrees of freedom that also are subject to lateral diffusion/Brownian motion in cell membrane.

The latter is characterized by a diffusion constant D, which typically lies for cell surface proteins in the range of 10^−9^ to 10^−11^ cm^2^/s,^10^ about 2–4 orders of magnitude lower than D = 4×10^−7^ cm^2^/s that has been measured for IgG in solution by Kihara et al.^11^ The average two-dimensional mobility of cell membrane proteins can be estimated according to Einstein^12^

(8) $$\bar{x^{2}}=2Dt$$

where $\bar{x^{2}}$ denotes mean square distance of displacement for particles undergoing Brownian motion, D is the diffusion coefficient and t is time. At D = 10^−10^ cm^2^/s, it will take ∼ 0.5 seconds for individual receptors to move on average ∼100 nm from their initial position. This period of time is short compared with the half-lives of typical antibody-antigen complexes that can reach into hours, and confirms that target cross-linking by a bispecific mAb is kinetically possible even when the average distances on the surface exceed the reach of the antigen-binding arms of the mAb. Combined with dimensional reduction, this means that the cross-linking reaction is very fast, ensuring that any monovalently bound bispecific antibody molecule quickly binds the other target too, if available. Effectively, the activity of a binding site depends on the state of the second site in the antibody molecule, i.e., they are not independent even if they are not expected to be directly interacting at all. As a result, no monovalently bound DuetMab was found by Mazor et al.,^5,6^ an observation similar to that made by Gavutis et al.^13^ for interferon binding to its heterodimeric receptor. In addition, even if a dissociation event does take place in a ternary complex, the species are still so close that the bond is likely to reform or another one to come by, depending on the relative speed of surface and volume reactions. Kinetically, this manifests as enhanced binding of an antibody through slower dissociation where half-lives are in hundreds of hours,^14^ which is commonly referred to as the avidity effect. We have shown here how the avidity effect can be accounted even when the epitopes are on freely moving non-interacting target molecules. It is sufficient for the targets to be anchored on the same cell membrane, and the epitopes do not necessarily need to reside on the same molecule or even on proteins within a stable target complex. We suggest that the same principle also applies to natural antibodies, of which the VkR95A variant DuetMab with its equal affinities for either target is a close analog on dual-positive cells.

A mechanistic model is usually an abstraction which reflects the modellers' interpretation of the information available for the system of interest and the kind of insight sought. To date, two approaches that aim to describe bispecific antibody interaction with two targets expressed on cell surface have been reported. First, Burke^15^ developed a more sophisticated model that also incorporates the targets' turnover and clearance pathways both for the free and bound versions of the drug. As for the drug-target interactions, those taking place between surface-bound species are defined though as if they were in solution, but with thousand-fold lower dissociation rate constants and 4-fold increased association rate constants. While superficially similar to the slower dissociation we predict for the cross-linked DuetMab, this approach contains an interesting paradox whereby the rate of the cross-linking reaction between membrane-bound species of defined surface concentration, say on a single cell, depends in the size of the volume in which this cell is suspended. Second, van Steeg et al.^16^ have adapted the avidity models developed by Kaufman and Jain^17^ and Müller et al.,^18^ for describing mAb binding in ELISA and BIAcore assays, respectively, to achieve good agreement between the model and data for mono- and -bispecific mAb binding to cells expressing EGFR and IGF1R targets. These fixed target position models calculate the fraction of any 2 receptors within the reach of the 2 binding sites of a mAb, defined as 49 nm in ref.^16^, 8.7 nm in ref.^17^ and 11 nm in ref.^18^ At receptor densities in excess of hundreds of thousands of molecules per cell, most of these can be calculated to lie within cross-linking distance of a mAb irrespective of the access radius value used, but, on H358 cells expressing 38260 copies EGFR and 23100 copies of IGF1R per cell,^16^ this fraction rises from 1% to around 30% when the access radius value is increased from 8.7 nm to 49 nm. No direct experimental data is available for EGFR and IGF1R, but in the case of cells expressing around 50000 copies of CD4 and CD70 each, all surface bound CD4/CD70-bispecific DuetMab antibody was found to be bivalently attached in saturating conditions,^5,6^ despite the average 49 nm distance between the receptors. Within the paradigm of the diffusion model, the higher effective antibody access radius values used in the fixed position model^16^ can be interpreted as accommodating the effect of the target lateral movement, while the empirically assigned volume of reaction V_r_ and surface association reaction rate constant adjustment parameter C_eff_ accommodate the two-dimensional surface reaction kinetics.

Our model has been developed with DuetMab-type bispecific mAbs that bind 2 different non-interacting targets in mind. Should the targets homo- and heterodimerize, as described by Mazor et al.^5^ for an anti-EGFR/HER2 DuetMab, the situation is more complex. If both epitopes on a heterodimer happen to be optimally placed, a target heterodimer might bivalently bind one DuetMab through both antigen-binding arms at the same time. Alternatively, if that is not possible for steric reasons or the rotation of the antigen-binding arms around the mAb hinge region is hindered, hetero-oligomerization is conceivable whereby DuetMab molecules alternate with target molecules in chain-like linear structures as discussed and modeled by Shea et al.^19^ Without prior knowledge of the location, the relative orientation of the epitopes and detailed structural information about the antibody-antigen complex, it will be difficult to decide which form of cross-linking will prevail. From the experimental point of view, any oligomeric antibody-antigen complexes could be detectable through single-molecule fluorescence microscopy.^20^ Therefore, an appropriate model of the formation of dimers or oligomers can be constructed and used in conjunction with the binding framework proposed in this model.

In the study reported here, we obtained similar results both by ODE and Monte Carlo modeling in conditions where both were applicable, but the two approaches are complementary and not identical. ODE simulations are much faster, and hence the only feasible approach for PK modeling, but they also assume the well-mixed approximation in the volume and on the surface. There is no such limitation for Monte Carlo simulations, and, albeit at heavy computational price, local concentration gradients or complex geometries can explicitly be taken into account. Alternatively, partial differential equation models should be applicable too. Likewise, while the size of the molecules is not explicitly defined in the ODE approach, this is essential for the Monte Carlo simulations within a MCell3 environment when chemical reactions are only allowed between molecules within reaction distance, here defined as the sum of respective hydrodynamic radiuses. On a separate note, the height of the cuboid volume with just 10-fold molar excess of the DuetMab in Monte Carlo models reached well beyond the distances encountered between neighboring cells in solid tissues, suggesting that local gradients may easily develop in such confined environments, i.e., well-stirred approximations might not be appropriate. These local gradients would be expected to be attenuated in vivo by the interstitial fluid flow that would replenish the antibody lost from the volume phase. The bulk flow, if present, effectively increases the volume available beyond the immediate thickness of the interstitial fluid layer the cell is exposed to.

Our model, though mechanistic, is an abstraction. The cell is presented as a sphere of 10 µm diameter that carries on its surface randomly placed non-interacting CD4 and CD70 target molecules. Both targets are subject to lateral Brownian motion defined by the diffusion coefficient assumed to be uniform across the entire surface of the cell. We appreciate that the 2D association rate constant estimated from the CD2-CD58 interaction that we used in the Monte Carlo model may itself be a composite parameter with fractional components both from diffusion and intrinsic chemical reaction steps. It was measured in a system where one of the reactants was embedded onto an artificial membrane where the lateral diffusion coefficients tend to be higher, i.e., the relative contribution of chemical reaction may be higher than when both reactants are linked to a genuine plasma membrane. Recently, Zheng et al.^21^ have noted an apparent increase in affinity of an EGFR/c-Met bispecific mAb for the second target that follows the binding of the first one, which in the diffusion model is accounted for through reduction in dimensionality of the surface association reaction, and hence the binding free energies in these two environments are not necessarily identical. The rate constants we used to model reactions taking place at 4°C were measured at 25°C, and are likely to be overestimates as both antibody-antigen association and dissociation rate constants decrease with temperature, as shown by Johnstone.^22^ Finally, our model does not include target-antibody complex internalization since, at the low incubation temperatures used by Mazor et al., this process is expected to be negligible. At physiological temperatures, the half-life of receptor-antibody complexes can vary from minutes to hours^23,24^ and, being significantly faster than dissociation from the surface-bound state, as shown on Fig. 6, would need to be included separately in the kinetic scheme. Despite these limitations, the surface diffusion model described can be experimentally verified and amended, when necessary, because it only uses experimentally measurable parameters with well-defined meaning. Not all parameters used in the model affect the results to the same extent. The BIAcore-measured rate constants are experimental values that describe the association and dissociation kinetics of mAbs, and are essential for the binding curve shown on Fig. 3A as at low DuetMab concentrations the level of binding appears kinetically limited. The 1–3 fold microscopic reversibility correction from Equations 8–10 made no discernible difference to the ODE simulation results, but was applied for the correctness, and any future adjustments, if necessary. Likewise, the two-dimensional chemical reaction step rate constant value for the Monte Carlo model is of limited consequence in the current model as long as it exceeds the rate of the diffusion step. The estimated value was implemented as it was required by the modeling environment. The diffusion-limited association rate constant value used in the ODE model for the surface-bound reactants accounts for the observed formation of cross-linked target species on double-positive target cells.

Mazor et al. demonstrated that a DuetMab,^5,6^ an IgG-like bispecific antibody that is monovalent for either antigen, can be engineered to be highly selective for the targeting of cell populations in complex mixtures according to the signature combinations of two surface-bound antigens. With the models described and made available here, we are adding *in silico* tools to analyze situations where the antibody affinities, receptor expression levels or accessibility can vary from one therapeutic target to another to further accelerate to process of drug discovery. For example, in the case of the recently Food and Drug Administration-approved combination therapy of cancer with two anti-CTLA4 and anti-PD1checkpoint inhibitor mAbs described by Mahoney et al.,^25^ the targets can be expressed on T cells found in blood, metastases or vascularized tumors, which all exhibit drastically different accessibility to antibody therapeutics, as summarized and quantitatively analyzed by Wittrup et al.^26^ Alternatively, if it is desirable to engage the targets also on single-positive cells, a combination of monospecific mAbs may be more appropriate since the avidity boosted binding to dual-positive cells would be lost on the target fraction where cross-linking is not possible, unless compensated by substantially improved monovalent binding affinity, which will take time and effort to develop.

In summary, we hope that the model presented here will be useful for accelerating the pace of drug discovery in the biopharmaceutical industry, and also provide a framework for the quantitative description of bivalent soluble ligand interaction with two different membrane-bound targets in general in systems immunology.

## Materials and methods

### Experimental bispecific mAb data

MFI data for DuetMab binding to target-expressing cells were all obtained from the paper by Mazor et al.^6^ using PlotDigitizer 2.6.8.^27^ Briefly, Mazor et al.^6^ incubated human T cells prepared from peripheral blood lymphocytes with a series of DuetMabs at desired concentrations for 1 h at 4°C, washed twice and stained for the bound antibody with a secondary fluorescently-labeled reagent for 45 min at 4°C. This was followed by two final washes and flow cytometric analysis for the bound IgG.

The MFI value measured in flow cytometry is expected to be proportional to the number of cell-bound DuetMab molecules. We used a conversion factor of 1.18 bound DuetMab molecules/MFI s assuming that, at 16 and 64 nM parent DuetMab concentrations, its binding to CD4+/CD70- cells is saturating (K_d_ = 0.9 nM). CD4 and CD70 expression levels on all three cell types were obtained from Mazor et al.^6^

### Kinetic scheme for the DuetMab interaction with two different targets expressed on the surface of the same cell

CD4 and CD70 are not known to have any binding affinity for each other, hence random initial distribution and no heterodimeric targets on the cell surface are assumed. Given that a trimolecular reaction between a solution-phase DuetMab and two cell membrane targets is extremely unlikely, we adopt a sequential mechanism shown in Fig. 1.

We use and compare 2 approaches for mathematical description of this kinetic scheme: an ODE model and a numerical Monte Carlo model.

### ODE model

The following system of two algebraic equations and three ODEs describes the reaction scheme outlined in Fig. 2 in conditions where the antibody concentration in solution is constant.

(1) $${\left[T_{1}\right]}_{s}={\left[T_{1}\right]}_{TOTs}-{\left[AT_{1}\right]}_{s}-{\left[AT_{1}T_{2}\right]}_{s}\;\;\;\text{and}\;{\left[T_{2}\right]}_{s}={\left[T_{2}\right]}_{TOTs}-{\left[AT_{2}\right]}_{s}-{\left[AT_{1}T_{2}\right]}_{s}$$

(2) $$\frac{d{\left[AT_{1}\right]}_{s}}{dt}=k_{1}\left[A\right]{\left[T_{1}\right]}_{s}-k_{-1}{\left[AT_{1}\right]}_{s}-k_{3}{\left[AT_{1}\right]}_{s}{\left[T_{2}\right]}_{s}+k_{-3}{\left[AT_{1}T_{2}\right]}_{s}$$

(3) $$\frac{d{\left[AT_{2}\right]}_{s}}{dt}=k_{2}\left[A\right]{\left[T_{2}\right]}_{s}-k_{-2}{\left[AT_{2}\right]}_{s}-k_{4}{\left[AT_{2}\right]}_{s}{\left[T_{1}\right]}_{s}+k_{-4}{\left[AT_{1}T_{2}\right]}_{s}$$

(4) $$\frac{d{\left[AT_{1}T_{2}\right]}_{s}\;}{dt}=k_{3}{\left[AT_{1}\right]}_{s}{\left[T_{2}\right]}_{s}-k_{-3}{\left[AT_{1}T_{2}\right]}_{s}+k_{4}{\left[AT_{2}\right]}_{s}{\left[T_{1}\right]}_{s}-k_{-4}{\left[AT_{1}T_{2}\right]}_{s}$$

In Equations 1–4 [A] denotes DuetMab molar concentration (M) in solution. The rest are cell surface-bound species in mol/dm^2^ units and marked with subscript *s*. ${\text{T}}_{1}$ and ${\text{T}}_{2}$ are free targets (CD4 and CD70, respectively), AT_1_ and AT_2_ are DuetMab complexes with one or the other target and ${\text{AT}}_{1}{\text{T}}_{2}$ is the ternary complex between two different targets cross-linked by one DuetMab molecule. Association rate constants *k*_*1*_ and *k*_*2*_ for reactions between a volume and a surface molecule are in 1/(M s) units, while *k*_*3*_ and *k*_*4*_ for reactions between two surface molecules are in dm^2^/(mol s) units. Dissociation rate constants *k*_*-1*_, *k*_*-2*_, *k*_*-3*_ and *k*_*-4*_ are all in 1/s units.

The initial conditions at t = 0 are defined as

(5) $${\left[T_{1}\right]}_{0s}={\left[T_{1}\right]}_{TOTs},\;\;{\left[T_{2}\right]}_{0s}={\left[T_{2}\right]}_{TOTs},\;\;{\left[AT_{1}\right]}_{0s}={\left[AT_{2}\right]}_{0s}={\left[AT_{1}T_{2}\right]}_{0s}=0$$

where ${\left[T_{1}\right]}_{TOTs}$ and${\left[T_{2}\right]}_{TOTs}$ correspond to the total concentrations of the surface targets assuming uniform distribution on the surface of a 10 µm diameter spherical cell. Equations 1–4 were implemented using the ode45 function in Matlab R2015b (The MathWorks Inc., Natick, MA, USA) and in volume concentration terms in Simbiology v5.2 (The MathWorks Inc., Natick, MA, USA) (Supplementary Data).

### Monte carlo (MC) simulation

Simulations were performed using MCell3 (Pittsburgh University Biological Supercomputing Center, USA),^28-31^ which is a computational biology application for Monte Carlo simulation of biological processes involving diffusion and chemical reactions. A virtual reaction vessel in the shape of a cuboid with square bottom surface area of 0.1 µm^2^ and variable height was defined in the MCell3 plug-in running in Blender 2.74.^32^ The bottom of the cuboid was seeded with the targets T_1_ and T_2_ for CD4 and CD70 at appropriate density for the cell type modeled assuming a sphere of 10 µm diameter (26 and 29 targets for CD4 and CD70 in dual-positive cells and 21 and 16, respectively, in single-positive cells). The height of the cuboid was adjusted to accommodate 605 randomly placed volume phase DuetMab molecules at the desired concentration at t=0 to keep the maximum reduction to volume phase DuetMab particles number below 10% (Table S1). 0.1–100 µs time steps and 3 parallel runs were used to simulate the chemical and diffusion processes on a HP Elitebook 8570 w. The rate and diffusion constant values are listed in Table 2.

**Table 2.** Target-related parameters. Receptor numbers are from Mazor et al.^6^

	CD4		CD70	
Parameter	Per cell	Per cuboid	Per cell	Per cuboid
Receptors on CD4+/CD70+ cells	82000	26	92000	29
Receptors on CD4+/CD70- cells	68000	22	0	0
Receptors on CD4−/CD70+ cells	0	0	55000	17
^1^Diffusion coefficient D, cm^2^/s	5×10^−10^		5×10^−10^	

1 CD4 subpopulations of different mobility, characterized by D=1.14×10^−8^ cm^2^/s, D = 2.34×10^−10^ cm^2^/s and D = 3.31×10^−11^ cm^2^/s were measured by Zhan et al.,^40^ D = 5×10^−10^ cm^2^/s according to Pal et al.,^41^ D= (1.87–20.9)×10^−10^ cm^2^/s and D = 1.35×10^−10^ cm^2^/s according to Rawat et al.,^43^ D=5×10^−9^ cm^2^/s according to Baker et al.^43^ and D = 2.7×10^−10^ cm^2^/s according to Finnegan et al.^44^ mAb solution diffusion coefficient D = 4×10^−7^ cm^2^/s has been reported by Saltzman.^45^

### Rate constant values

Volume phase rate constants *k*_*1*_, *k*_*-1*_, *k*_*2*_ and *k*_*-2*_ for DuetMab interaction with CD4 or CD70 were obtained from the article by Mazor et al.,^6^ who measured them in vitro using surface plasmon resonance. No experimental data is available for an anti-CD4/CD70 DuetMab binding to its respective antigens when both reactants are surface-bound for the values of *k*_*3*_, *k*_*-3*_, *k*_*4*_ and *k*_*-4*_. However, *k*_*1*_ and *k*_*2*_ are close to each other for all DuetMabs studied and within the range that is typical for antibody-antigen interactions of 10^5^-10^6^ 1/(M s),^33^ which is considered to be diffusion-dominated in solution.^34^ Given that protein diffusion coefficients in cell membrane are 3–4 orders of magnitude smaller than for IgG in solution (Table 2), we postulate that association reaction constants k_3_ and k_4_ are equal and diffusion-dominated also on the cell surface.

According to Torney and McConnell^35^ and Goldstein,^36^ the two-dimensional diffusion-limited association reaction constant $k_{on}$ expressed per molecule is proportional to the sum *D* of the reactants' surface diffusion coefficient values

(6) $$k_{on}=4D$$

A number of surface diffusion coefficient values have been measured for CD4, as listed under Table 2, of which we adopt the median at ∼ 5×10^−10^ cm^2^/s. No such value is available for CD70, but, given that it has a single 21–22 amino acid long α-helical transmembrane domain similar to that of CD4, we assume that their diffusion coefficients are similar. If so, the diffusion-limited 2D surface association reaction rate constant in dm^2^/mole/s units for the ODE-based model is defined as

(7) $$k_{3}=k_{4}=2.4\times 10^{13}{\text{dm}}^{2}/\left(\text{mol}\;\text{s}\right)$$

According to the principle of general microscopic reversibility,^37^ the rate constants for the reaction scheme given on Fig. 2 are related according to Equation 8

(8) $$k_{1}k_{-2}k_{3}k_{-4}=k_{-1}k_{2}k_{-3}k_{4}$$

With the volume reaction rate constants *k*_*1*_, *k*_*−1*_, *k*_*2*_ and *k*_*−2*_ measured independently and *k*_*3*_* = k*_*4*_, this allows the relation of the surface dissociation constants *k*_*−3*_ and *k*_*−4*_ to the volume reaction constants *k*_*1*_, *k*_*−1*_, *k*_*2*_ and *k*_*−2*_

(9) $$\frac{k_{-4}}{k_{-3}}=\frac{k_{-1}k_{2}}{k_{1}k_{-2}}$$

The dissociation constant for a reaction taking place on the surface does not need to be identical to that taking place in solution, but can be expected to be related, and the absolute values in surface and volume concentration conditions for interferon dissociation from its receptor were only 3-fold different when measured by Gavutis et al.^13^ This effect is relatively small and we hence assume $k_{-3}=k_{-2}$ for the DuetMab invariant interaction with CD70. If so, the surface dissociation rate constant $k_{-4}$ for CD4 can be found from its volume dissociation rate constant value $k_{-1}$ according to Equation 10. It should be noted that the correction factor *k*_*2*_*/k*_*1*_ only varies from 0.9 to 2.5, and the choice between assuming either *k_−2_* = *k_−3_* or *k_−1_ = k_−4_* would not affect the simulation results.

(10) $$k_{-4}=k_{-1}\frac{k_{2}}{k_{1}}$$

The MCell Monte Carlo model requires a distinct surface 2D chemical reaction association rate constant value in addition to the reactants' surface diffusion coefficients. We use 0.7 µm^2^/(molecule s) (equivalent to 4.2×10^13^ dm^2^/(mol*s) that we calculated from the K_d_≈6 molcules/µm^2^ surface dissociation equilibrium constant and the dissociation rate constant k_off_ = 5 1/s measured for the interaction between membrane-bound forms of CD2 and CD58 by Zhu et al.^38^ and van der Merwe et al.,^39^ respectively. In solution, this protein-protein interaction displays a typical antibody-antigen association rate constant k_on_ = 2×10^5^ 1/(M s).^39^

DuetMab binding to single-positive cells is monovalent, and there are no differences between the MFI values for variants with K_d_ > 25 nM. This suggests that these surface-bound DuetMabs, with dissociation half-lives in the minute range or less (Table 1), are lost during the final cell washing steps before flow cytometry. We therefore corrected the model estimates for DuetMabs bound to single-positive CD4^+^/CD70^−^ and CD4^−^/CD70^+^ cells shown on Figs. 2 and 3 according to the first-order exponential decay function (11) using rate constants k_−1_ and k_−2_ for the appropriate DuetMab variant from Table 1.

(11) $$[AT_{1}]={\left[AT_{1}\right]}_{c}e^{-k_{-1}t}\;\;[AT_{2}]={\left[AT_{2}\right]}_{c}e^{-k_{-2}t}$$

where ${\left[AT_{1}\right]}_{c}$ and ${\left[AT_{2}\right]}_{c}$ are the complex concentration value calculated for one-hour and $k_{off}$ is the dissociation rate constant k_−1_ or k_−2_ for the relevant DuetMab-antigen binary complex. We assume t = 240 s (approximate time required for two spin-resuspend cycles and rapid flow cytometric analysis). We do not attempt to quantify in our model the residual experimental non-specific binding of low affinity DuetMabs at higher antibody concentrations, which appears to reach ∼15% of the total on single-positive cells.

## Supplementary Material

KMAB_A_1178437_supplemental_material.zip
