# Supplementary material for: Modeling bispecific monoclonal antibody interaction with two cell membrane targets indicates the importance of surface diffusion
Source: MAbs. 2016 Apr 20;8(5):905–15. doi: 10.1080/19420862.2016.1178437 (PMC4968105; doi:10.1080/19420862.2016.1178437)
Supplement: KMAB_A_1178437_supplemental_material.zip [file kmab-08-05-1178437-s001.zip › Diffusion bispecific GSK Supplementary_Review.docx]

**Supplementary Information**

**Modelling bispecific monoclonal antibody interaction with two cell membrane targets indicates the importance of surface diffusion**

Bram G. Sengers, Sean McGinty, Fatma Z. Nouri, Maryam Argungu, Emma Hawkins, Aymen Hadji, Andrew Weber, Adam Taylor, Armin Sepp

***ODE model Matlab variant of the main text equations 1-4 in surface concentration units for surface-bound species and volume concentration for the antibody.***

Function ‘**runabASv8.m**’ defines the three cell types, cycles through them for each of the six DuetMab variants and stores the results in surface concentration units. Function ‘**kineticodefile.m**’ solves the ODEs for the time span defined. The results are obtained in surface concentration units. The MFI values are obtained by multiplying the respective surface concentration values with cell surface area and the Avogadro number to get the bound antibody numbers per cell and dividing the resulting value by 1.18 conversion factor.

***ODE model SimBiology variant of the main text ODEs 2-4 in volume concentration units***

The main text ODEs (2-4) define the reaction kinetics in two-dimensional concentration units. For the future application to PK/PD modelling purposes where volume concentrations are used, we converted these equations into molar concentration units. First, the main text ODEs (2-4) are multiplied by constant S/V where S is the surface area confined in volume V. This converts all surface concentrations in all terms except $k_{3}\left[ AT_{1} \right]_{s}\left[ T_{2} \right]_{s}$ and $k_{4}\left[ AT_{2} \right]_{s}\left[ T_{1} \right]_{s}$ where $\left[ T_{2} \right]_{s}$ and $\left[ T_{1} \right]_{s}$ remain, respectively. The surface concentration $\left[ T_{2} \right]_{s}$ and $\left[ T_{1} \right]_{s}$ in these terms are obtained by multiplying their respective volume concentrations $\left[ T_{2} \right]$ and $\left[ T_{1} \right]$ with V/S. The main text algebraic equations (1) were replaced with an ODE for the volume concentration of the mAb, yielding ODEs S1-S4.

$$\frac{d[A]}{dt}=-k_{1}[A]\left[ T_{1} \right]+k_{-1}\left[ AT_{1} \right]-k_{2}[A]\left[ T_{2} \right]+k_{-2}\left[ AT_{2} \right] (S1)$$

$$\frac{d\left[ AT_{1} \right]}{dt}=k_{1}[A]\left[ T_{1} \right]-k_{-1}\left[ AT_{1} \right]-k_{3}\left[ AT_{1} \right]\left[ T_{2} \right]\frac{V}{S}+k_{-3}\left[ AT_{1}T_{2} \right] (S2)$$

$$\frac{d\left[ AT_{2} \right]}{dt}=k_{2}[A]\left[ T_{2} \right]-k_{-2}\left[ AT_{2} \right]-k_{4}\left[ AT_{2} \right]\left[ T_{1} \right]\frac{V}{S}+k_{-4}\left[ AT_{1}T_{2} \right] (S3)$$

$$\frac{d\left[ AT_{1}T_{2} \right]}{dt}=k_{3}\left[ AT_{1} \right]\left[ T_{2} \right]\frac{V}{S}-k_{-3}\left[ AT_{1}T_{2} \right]+k_{4}\left[ AT_{2} \right]\left[ T_{1} \right]\frac{V}{S}-k_{-4}\left[ AT_{1}T_{2} \right] (S4)$$

In SimBiology model cell type A is double-positive CD4^+^/CD70^+^, types B and C are single-positive CD4^+^/CD70^-^ and CD4^-^/CD70^+^ respectively. Numbers 1-6 denote the DuetMab variants from the parent to the weakest binding one respectively as listed in Table 1 of the main text.

Task ‘**Binding time course’** simulates DuetMab binding kinetics.

Task ‘**Dissociation time course’** simulates DuetMab dissociation kinetics.

Task ‘**Scan binding**’ simulates DuetMab binding endpoints achieved for a given incubation time.

B-CD4, R-CD70, AB and AR- DuetMab in complex with CD4 and CD70 respectively, ABR-DuetMab cross-linking both targets.

Variants A1-A6,B1-B6 and C1-C6 denote DuetMab variants 1-6, as listed in Table 1 of the main text on double-positive CD4+/CD70+ and single-positive CD4+/CD70-and CD4-/CD70+ cells respectively.

The results are obtained in molar concentration units. By default, a single cell is suspended in 1 L volume at constant antibody concentration. At these settings the MFI value is obtained by multiplying the respective species volume concentration with Avogadro number and dividing the resulting value by 1.18 conversion factor.

***Monte Carlo model***


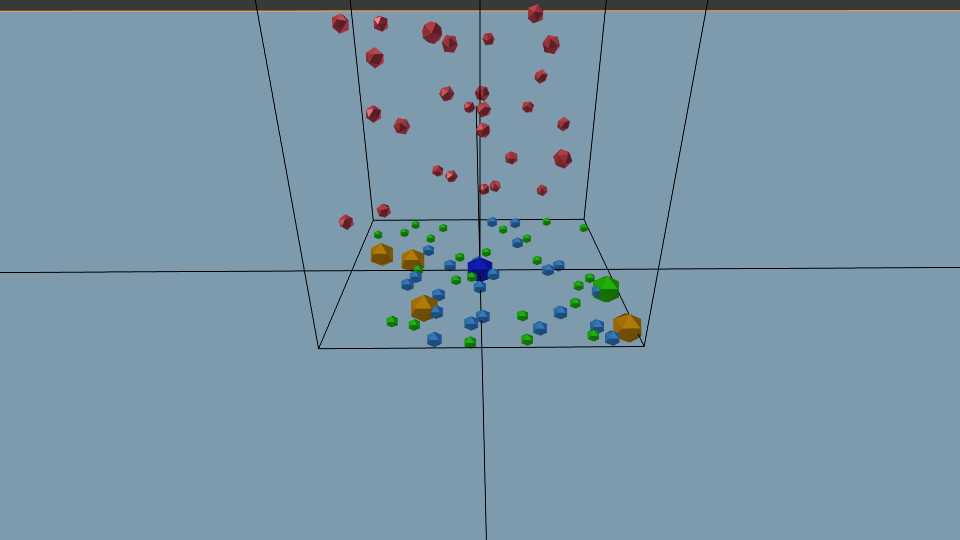


Supplementary Figure 1. MCell3 model “CD4+70+ 1E-06M Parent DuetmAb.blend” used for the simulation of DuetMab binding to cell surface expressed CD4 and CD70. Only the cuboid was defined in the model, the sphere is for illustration purposes only. Red particles: DuetMab molecules in solution. On the bottom surface representing the cell membrane there are small green particles for T_1_ (free CD4), or blue ones for T_2_ (free CD70), large green ones for AT_1_ (CD4-DuetMab), or blue ones for AT_2_ (CD70-DuetMab) and large yellow ones for AT_1_T_2_ (CD4/CD70-DuetMab tertiary complex). Screenshot taken for the CD4+/CD70+ double-positive cell at 1000 nM DuetMab concentration at t=0.43 sec. Cuboid dimensions are 0.31×0.31×10 µm^3^ with the height extending beyond the top of the image).

All surface molecules were confined to the bottom inside surface of the cell. The side walls of the cuboid are reflective to confine the surface-bound species to the bottom of the cuboid and the DuetMab molecules to the inside of the cuboid.

MCell3 (Pittsburgh University Biological Supercomputing Centre) [1-4], is a computational biology simulation application used as a plug-in for Blender v2.74 3D visualization and animation software suite [5]. The MCell3 version “**cellblender_v1.0_RC3**” used is attached for convenience as the model is not compatible with the the later version.

Table S1. Monte Carlo simulation settings. The rate and diffusion constants are listed in the main text.

| **DuetMab** | **iterations** | **dt (s)** | **Time simulated** | **Cuboid height (units/µm)** |
| --- | --- | --- | --- | --- |
| 1E-11M | 3.6e7 | 1e-4 | 1h | 1000000 |
| 1E-10M | 3.6e7 | 1e-4 | 1h | 100000 |
| 1E-09M | 3.6e7 | 1e-4 | 1h | 10000 |
| 1E-08M | 1e7 | 1e-4 | 1000 sec | 1000 |
| 1E-07M | 1e6 | 1e-4 | 100 sec | 100 |
| 1E-06M | 1e6 | 1e-5 | 10 sec | 10 |
| 1E-05M | 1e5 | 1e-5 | 1 sec | 1 |
| 1E-04M | 1e5 | 1e-5 | 1 sec | 0.1 |
| 1E-03M | 1e5 | 1e-6 | 0.1 sec | 0.01 |
| 1E-02M | 2e4 | 1e-6 | 0.02 sec | 0.001 |
| 1E-01M | 2e4 | 1e-7 | 0.002 sec | 0.0001 |

**References**

1. Kerr RA, Bartol TM, Kaminsky B, Dittrich M, Chang J-CJ, Baden SB, Sejnowski TJ, Stiles JR (2008) Fast Monte Carlo Simulation Methods for Biological Reaction-Diffusion Systems in Solution and on Surfaces. SIAM Journal on Scientific Computing 30 (6):3126-3149.

2. Stiles JR, Bartol TM (2001) Monte Carlo methods for simulating realistic synaptic microphysiology using MCell. In: De Schutter E (ed) Computational neuroscience. Realistic modeling for experimentalists. CRC Press, Boca Raton, FL.

3. Stiles JR, Van Helden D, Bartol TM, Jr., Salpeter EE, Salpeter MM (1996) Miniature endplate current rise times less than 100 microseconds from improved dual recordings can be modeled with passive acetylcholine diffusion from a synaptic vesicle. Proc Natl Acad Sci U S A 93 (12):5747-5752.

4. MCell Monte Carlo Cell. <http://www.mcell.org/index.html>.

5. Community BO (2015) Blender - a 3D modelling and rendering package. Blender Foundation.
